# Evidence-based budgeting policy in maternal and child health programme: do they work?

**DOI:** 10.1186/1471-2458-12-S2-A31

**Published:** 2012-11-27

**Authors:** M Faozi Kurniawan, Deni Harbianto, Digna Purwaningrum, Tiara Marthias

**Affiliations:** 1 Center for Health Service Management, School of Medicine, Gadjah Mada University, Jogjakarta, Indonesia

## Background

Despite Indonesia’s health status improvement over the last decade, special efforts to achieve the MDGs goals are still warranted, especially in maternal, neonatal, and child health (MNCH). However, the current centrally driven MNCH policies do not address the geographical disparities and the different constraints faced by Indonesian provinces and districts. Moreover, the MNCH slow progress may have been hindered by various funding constraints, for example small local budget allocation specifically for MNCH, lack of evidence-based budget planning leading to unsound health planning and implementation. Therefore, this study aims to assess the current health financing mechanism used in one of Indonesian Province of Papua, that has low MNCH outcome.

## Methods

This was an observational study using mixed-methods. Study subjects were from the Provincial and four selected District Health Offices in Papua Province of Indonesia. overnment official documents analysis and direct observations on the study subjects were done to assess the financing and budgeting for MNCH in Papua.

## Results

The study shows a low commitment from the local government in MNCH priority areas. The main reason was that the largest share of MNCH funding still comes from Central Government (ABPN), whereas the proportion funded from local sources is relativity low. Furthermore, there is a very limited use of evidence-based financing and budgeting, mainly due to limited capability of the human resources for health as well as the largely undocumented epidemiological and health system data. This limited human resource capability also largely affected by the low commitment of the local government.

## Conclusion

Despite the relatively large amount of MNCH funding in Papua, human resource limitation poses a serious problem in scaling up for priority interventions. Low local government commitment is still the main obstacle in health budgeting policy. These problems may also applicable to other districts of Indonesia and as unanticipated effects from ill-designed health decentralization.

